# Selective area multilayer graphene synthesis using resistive nanoheater probe

**DOI:** 10.1038/s41598-023-34202-y

**Published:** 2023-05-17

**Authors:** Ingrid Torres, Sadegh Mehdi Aghaei, Nezih Pala, Angelo Gaitas

**Affiliations:** 1 Department of Electrical and Computer Engineering, Florida International University, Miami, FL 33172 USA; 2 Department of Mechanical Engineering, Worcester Polytechnic Institute, Worcester, MA 01609 USA; 3 Icahn School of Medicine at Mount Sinai, New York, NY 10029 USA

**Keywords:** Synthesis of graphene, Graphene

## Abstract

Graphene has been a material of interest due to its versatile properties and wide variety of applications. However, production has been one of the most challenging aspects of graphene and multilayer graphene (MLG). Most synthesis techniques require elevated temperatures and additional steps to transfer graphene or MLG to a substrate, which compromises the integrity of the film. In this paper, metal-induced crystallization is explored to locally synthesize MLG directly on metal films, creating an MLG-metal composite and directly on insulating substrates with a moving resistive nanoheater probe at much lower temperature conditions (~ 250 °C). Raman spectroscopy shows that the resultant carbon structure has properties of MLG. The presented tip-based approach offers a much simpler MLG fabrication solution by eliminating the photolithographic and transfer steps of MLG.

## Introduction

Graphene has emerged as one of the most promising materials for the post-silicon era^1^. The simplest method to obtain graphene is through exfoliation, which involves peeling carbon layers from graphite until a monolayer or few layers are obtained^1^. However, the exfoliation technique is very time-consuming. Another common method is Chemical Vapor Deposition (CVD), used for graphene synthesis, which can produce large-area graphene layers^2,3^, but it is performed at high temperatures (> 950 °C). In addition, it requires the mechanical transfer of graphene to other surfaces for further processing. These additional steps introduce impurities, defects, tears, and wrinkles, dramatically dampening the properties of graphene^4–7^. Therefore, low-temperature and transfer-less graphene synthesis is highly desirable for large-area industrial applications, particularly to conserve the mechanical integrity of the low dielectric constant of intermetal dielectrics commonly used in integrated circuit (IC) fabrication^8–10^.

Multilayer Graphene (MLG) is an excellent option for wiring and electrodes in applications requiring high electrical/thermal conductivities. Graphene synthesis with a controllable number of layers has been possible by controlling the thickness of the evaporated amorphous carbon layer and inducing metal-catalyzed crystallization at 650–950°C^11^. When heated, the carbon atoms diffuse into the metal and precipitate at the surface while cooling down^11^. MLG has also been reported to form at the interface between the metal and the substrate at 800°C^12^. This exchange between a metal catalyst and a group IV material is known as Layer Exchange (LE)^13^. Metal-Induced Crystallization (MIC) is a simple and effective method to lower the synthesis temperature of MLG directly on substrates by inducing the LE. Synthesis occurs directly on the substrate, eliminating additional steps of mechanically transferring MLG. Tin (Sn) has the advantage over other metals of allowing synthesis at the surface of the metal and on the insulating substrate at 250 °C due to its low melting point^14^.

In this work, we directly synthesize MLG on Sn and on the insulating substrate at a low temperature (≈250 °C) using the MIC-LE^14^. Rather than heating the entire sample, localized heating at a desired location on the sample is provided with a resistive nanoheater probe tip. Nano-heater tip-based methods have been used to reduce insulating graphene oxide films (GO) to locally create graphene^15–17^; however, this is the first time tip-based heating is used with MIC-LE for MLG synthesis. This new method is compatible with existing fabrication processes and has the potential for expansion to high throughput applications^18–22^. The direct, transfer-less, and mask-less synthesis of MLG on metals and insulating substrates achieved in this work enables the integration of MLG with Complementary Metal-Oxide Semiconductor (CMOS) processing^23^.

## Methodology

**Figure 1:**
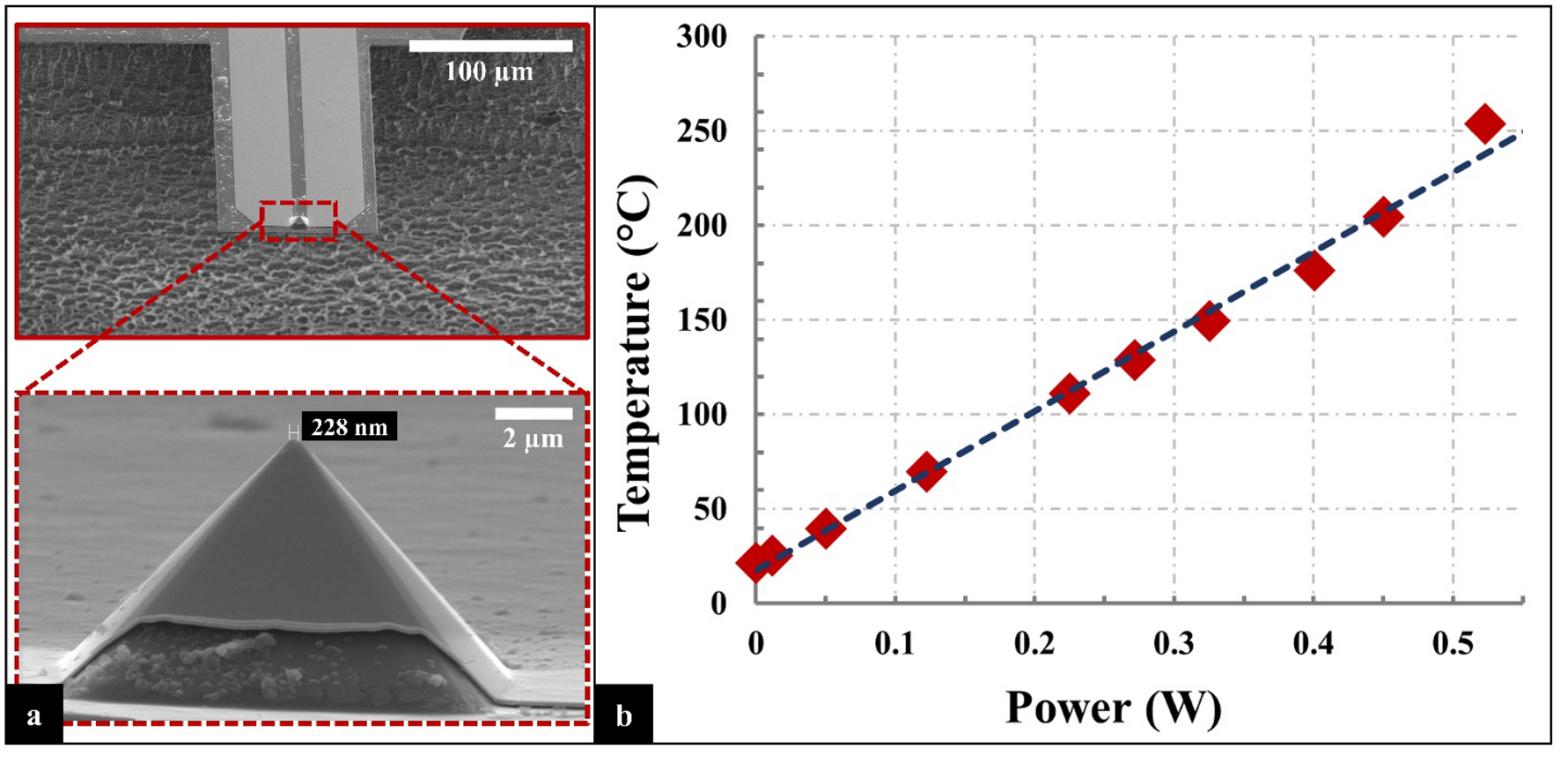
(**a**) SEM images of the fabricated resistive nanoheater probe. (**b**) The temperature as a function of the input power.

**Figure 2:**
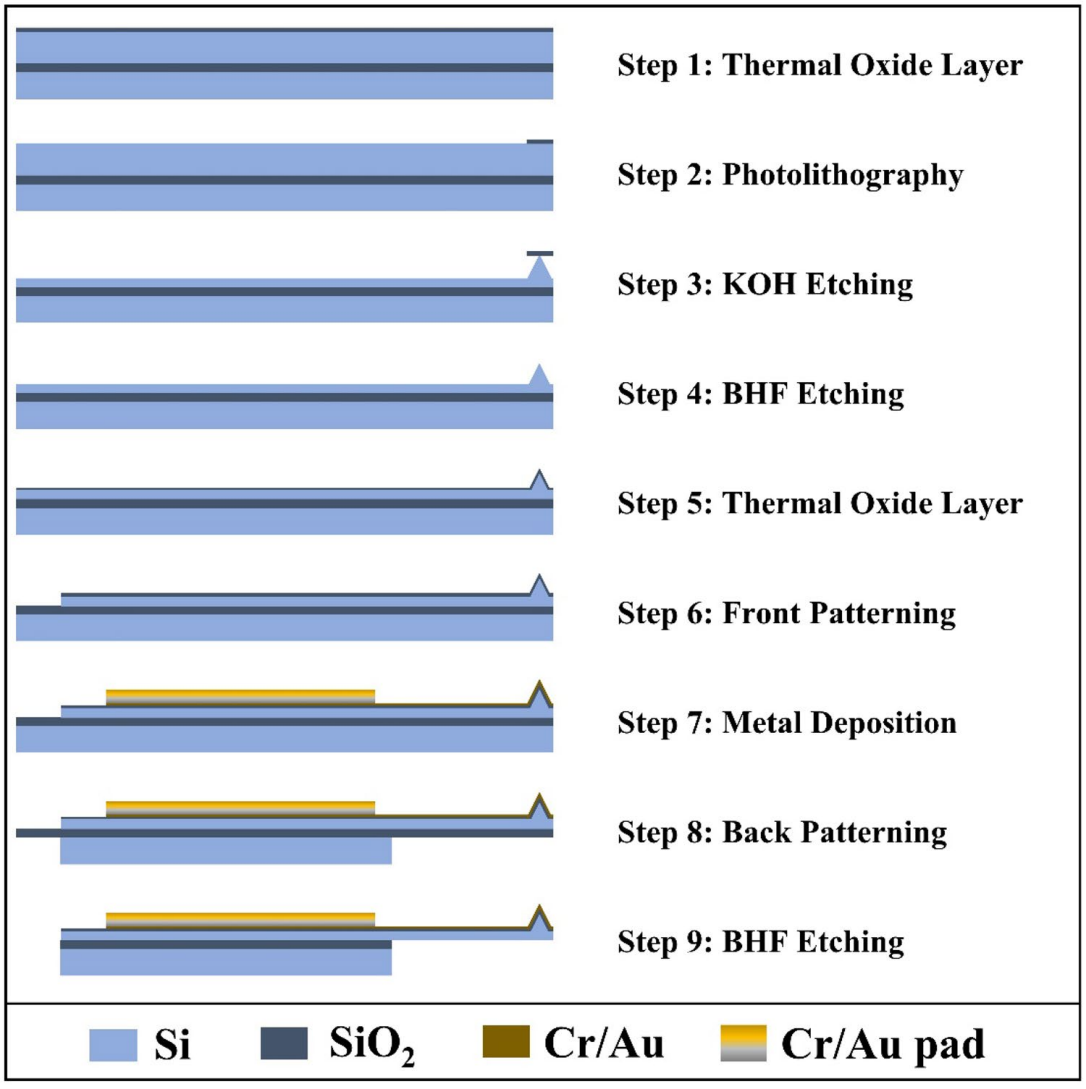
Nanofabrication steps of the resistive nanoheater probe.

A resistive nanoheater probe (Fig. 1a) is used to perform direct graphene synthesis by locally heating the sample at a predetermined area^24^. The fabrication process for the resistive nanoheater probe, shown in Fig. 2 (with corresponding mask layouts/top views in Supplementary Information, Fig. S.1), starts with an SOI wafer with 1 µm SiO_2_ buried oxide layer thickness and a 10 µm Si device layer. A 300 nm thermal oxide layer is deposited (Step 1) and patterned through photolithography (Step 2). Next, potassium hydroxide (KOH) is used for wet anisotropic etching to form the tip (Step 3). The oxide layer is etched away with Buffered Hydrofluoric Acid (BHF) (Step 4). A 100 nm thermal oxide layer is grown (Step 5). The cantilever is front patterned using Deep Reactive Ion Etch (DRIE) (Step 6). Then, metal deposition is performed by evaporating 10 nm/100 nm thick Cr/Au to form the pads and heaters (Step 7). The cantilever is formed by performing photolithography and patterning the back of the substrate through DRIE, where the buried oxide layer acts as an etch-stop layer (Step 8). Lastly, the probe is released by etching the buried oxide layer with BHF (Step 9).

The cantilever contains a microheater element made of the Cr/Au located at the tip with a nominal resistance of ≤ 10 Ohms. The tip height is 7.75 µm with a diameter of ≈200 nm. The rectangular cantilever is 100 µm wide, 150 µm long, 2 µm thick and rests on a 3 mm × 1.4 mm × 0.5 mm chip (Fig. 1a). The chip is glued on a probe board, then two thin copper cables are pasted on each pad with conductive epoxy and cured in the oven at 90 °C for 20 min.

To calibrate the resistive nanoheater probe, a 12 µm thermocouple (CHAL0005, Omega Engineering, Norwalk, CT, USA), as shown previously^22,24,25^, is brought into contact with the tip of the probe (Shown in Supplementary Information, Fig. S.2) while a source meter (Keithley 2400, Tektronix, Inc., Beaverton, OR, USA) is used to supply voltage to the resistive nanoheater probe in order to slowly increase the temperature at the tip of the cantilever. Temperature, as a function of the resistive nanoheater probe input power, is recorded (Fig. 1b). At ~ 520 mW, the temperature reaches 250 °C.

A system that includes a custom-made glass chamber with a 12″ diameter and 12″ height was modified for these experiments^25^ to include a flat optical window (detailed in the Supplementary Information, Fig. S.3). Through the window, the movement of the sample and the tip of the probe using a microscope is monitored. The chamber contains four fitted connectors. One connector is used for gas flow and to depressurize the chamber through a vent. A pressure gauge is placed on a second connector to monitor the pressure of the chamber. The third connector is used for mechanical pumping > 1 × 10^–3^ Torr, and the last connector is used for the wiring between the motorized stage and the probe to the external power supplies and a computer. The chamber and the interior parts are placed on top of a vibration isolation table. The parts inside the chamber consist of a fixture used to hold the probe in place and a stage capable of moving in the X, Y, and Z directions. The stage is composed of two mechanisms, the main platform for long-range motion (KT-LS28-MV, Zaber, Vancouver, Canada) and a second platform for short-precise motion (Tritor 100-XYZ, Piezosystem Jena, Germany). Because of the enclosed environment, the platforms are interfaced with a computer that uses a LabView program (NI, Austin, TX), which is used to move the stages. Through the LabView software, the platforms are moved to position the sample under the probe and brought into contact with the tip at a chosen location, while monitoring with the optical microscope. A source meter, Keithley 2400, is used to heat the nanoprobe when in contact with the sample.

**Figure 3:**
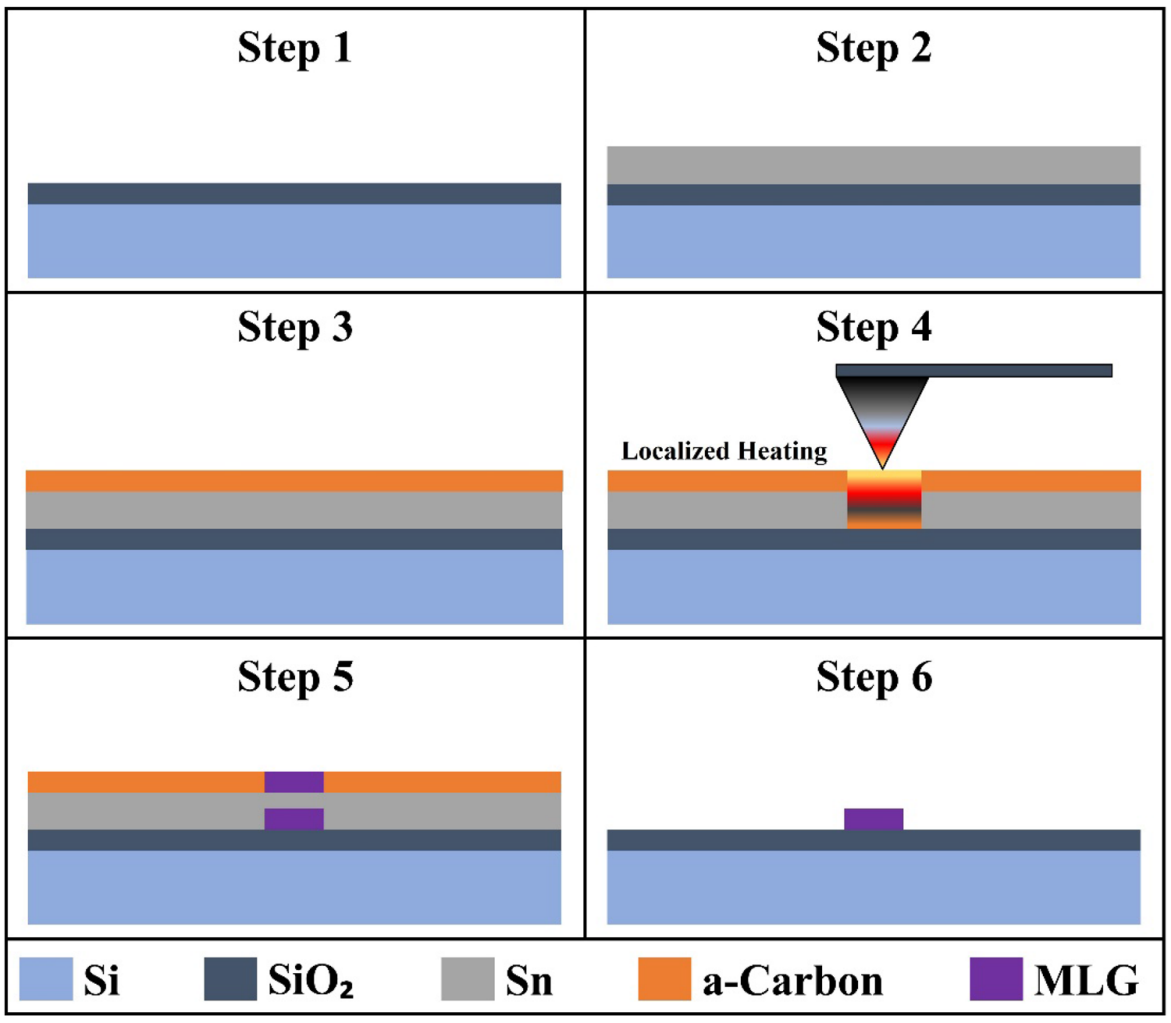
Schematic diagram of in-situ MLG synthesis.

The step-by-step process of the synthesis experiment is shown in Fig. 3. The sample consists of a 10 mm × 10 mm Si/SiO_2_ die, with a SiO_2_ thickness of 300 nm (Step 1). First, 500 nm thick Sn is evaporated on top of the substrate with an E-beam evaporator (CHA SAP-600, CHA Industries, Fremont, CA, USA) (Step 2). This is followed by sputtering of a-C using a sputter coater (Pelco SC-7, Ted Pella Inc., Redding, CA, USA) for 60 nm thickness (Step 3). Next, the sample is placed on the system’s platform, and the probe is positioned above the surface of the sample at an approximate distance of < 3 mm. The bell jar encloses the probe and the platform, and the pressure inside the chamber is reduced below 1 Torr (as seen in the homemade system schematic in the Supplementary Information, Fig. S.3). Once the base pressure is achieved, argon gas flows at a rate of 100–130 sccm, increasing the pressure to around 4 Torr. After the pressure in the chamber stabilizes, the MLG synthesis experiment is initiated by locally heating a specific area of the sample using the resistive nanoheater probe. Using LabView, the main platform is moved close to the surface of the sample. Then, the secondary platform controlled by the Piezosystem is moved to carefully bring the sample in contact with the probe (Step 4).

After the tip makes contact with the surface of the sample, the source-meter supplies voltage to the nanoheater to resistively heat it to 250 °C for a predetermined amount of time. The low melting point of Sn, 231.9 °C, has proven to be effective for MLG synthesis^14^. When the Sn/a-C is locally heated, the a-C dissolves and the Sn melts, causing the carbon atoms to diffuse into the metal. The carbon atoms segregate on the surface of the metal when the concentration of the atoms is supersaturated, forming graphene layers above and below the metal (Step 5). Finally, the sample is dipped in 10% Nitric Acid (HNO_3_) to remove the metal and lift the carbon structure in order to leave the MLG structure below (Step 6).

The characterization of all the samples is performed using Raman spectroscopy (Renishaw InVia, UK) with a laser of HeNe and 633 nm wavelength. Lorentzian function modeling is performed on the Raman spectra for peak fitting to estimate the Full Width at Half Maximum (FWHM). Scanning Electron Microscopy (SEM) – Energy Dispersive Spectroscopy (EDS) (JSM-F100, Jeol, Tokyo, Japan) is used for chemical compositional analysis at an acceleration voltage of 5 kV.

## Results and discussion

The position, shape, intensity of prominent peaks, and intensity ratios of the Raman spectra provide a recognizable signature for the positive identification of synthesized MLG^26–28^. Our initial Raman analysis of the sample detects a broad peak around 1500 cm^−1^, confirming sputtered a-C film on top of Sn prior to heating^27^ (See Supplementary Information, Fig. S.4a).

**Figure 4:**
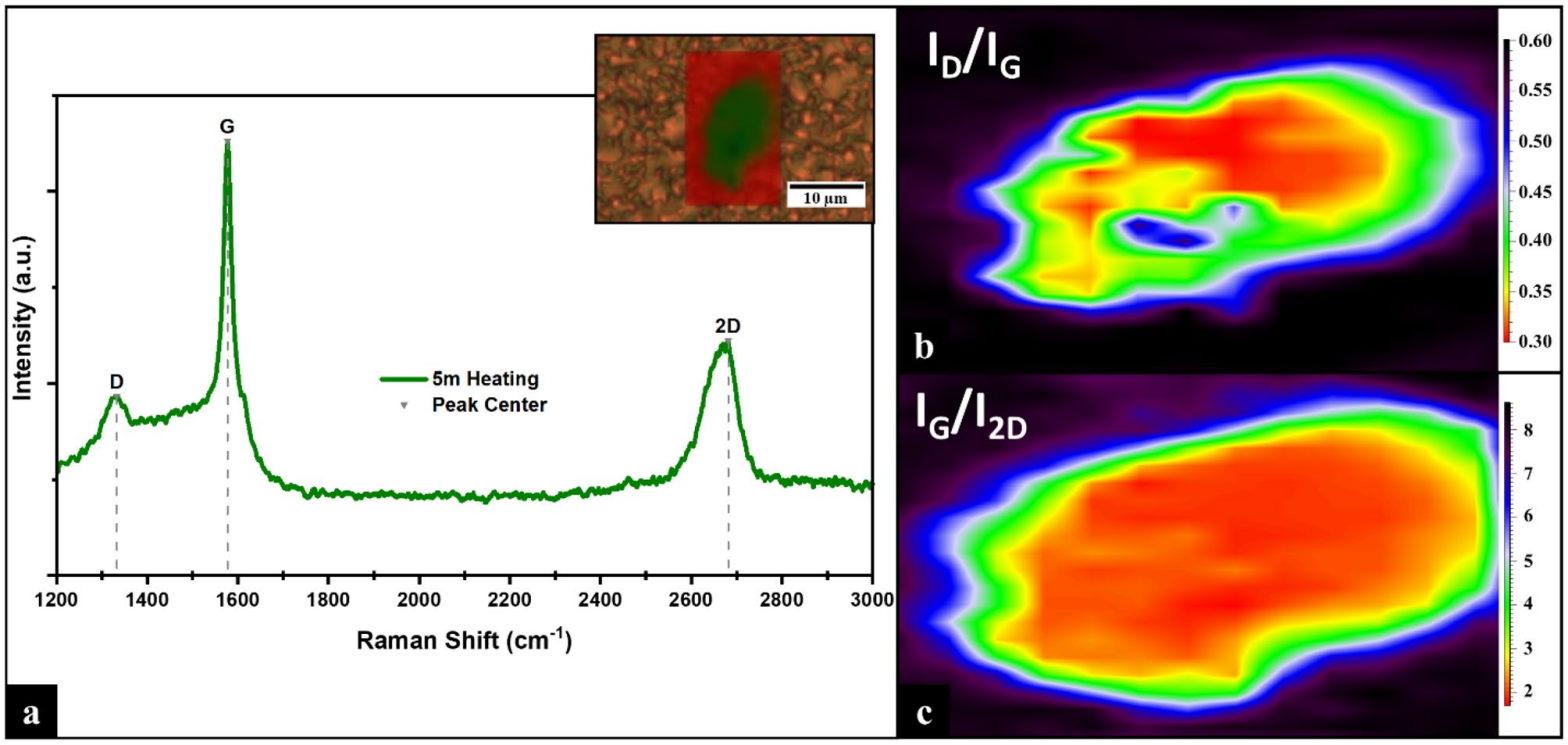
(**a**) Raman spectrum of the sample when heated for 5 min with inset of the surface area composition, a-C in red and MLG in green. (**b**) Raman I_D_/I_G_ ratio map. (**c**) Raman I_G_/I_2D_ ratio map.

The Raman spectrum at 5 min of heating time (Fig. 4a) shows the characteristic D band at 1333 cm^-1^, G band at 1577 cm^-1^, and 2D band at 2681 cm^-1^, indicating graphene formation^3,26,29–33^. The G and 2D peaks are well-defined narrow, sharp peaks with FWHM of 30 cm^-1^ and 79 cm^-1^, respectively, and a ratio I_G_/I_2D_ of 1.85. For CVD-graphene, the I_G_/I_2D_ ratio and the shape of the 2D peak provide an estimate of the number of graphene layers present on the structure^3,26,33,34^. Comparing the obtained Raman spectra with results reported for CVD graphene indicates that the synthesized structure is MLG of 3 to 5 layers^3,26,29,33^. The D peak has a FWHM of 100 cm^-1^ and an I_D_/I_G_ ratio of ~ 0.41. The crystal quality of the MLG is defined by the G-to-D peak intensity ratio, which is proportional to the number of defects^34–36^. The value of 0.41 indicates high-quality MLG with low defect density^33,36^.

Raman surface area map analysis (Fig. 4a Inset) shows regions of a-C (red) and the region of MLG (green) with a length of ≈13.567 µm and an area of ≈88.867 µm^2^﻿. The Raman I_D_/I_G_ ratio map (Fig. 4b) indicates a large area of high quality MLG where the ratio ranges between 0.30 and 0.45. The Raman I_G_/I_2D_ ratio map (Fig. 4c) shows the lowest values at the central area of the structure, representing the least number of graphene layers. Beyond 5 min of heating, unwarranted defects, disorder, and damages started to form (Supplementary Information, section “Raman Spectra Evolution” and Fig. S.4).

**Figure 5:**
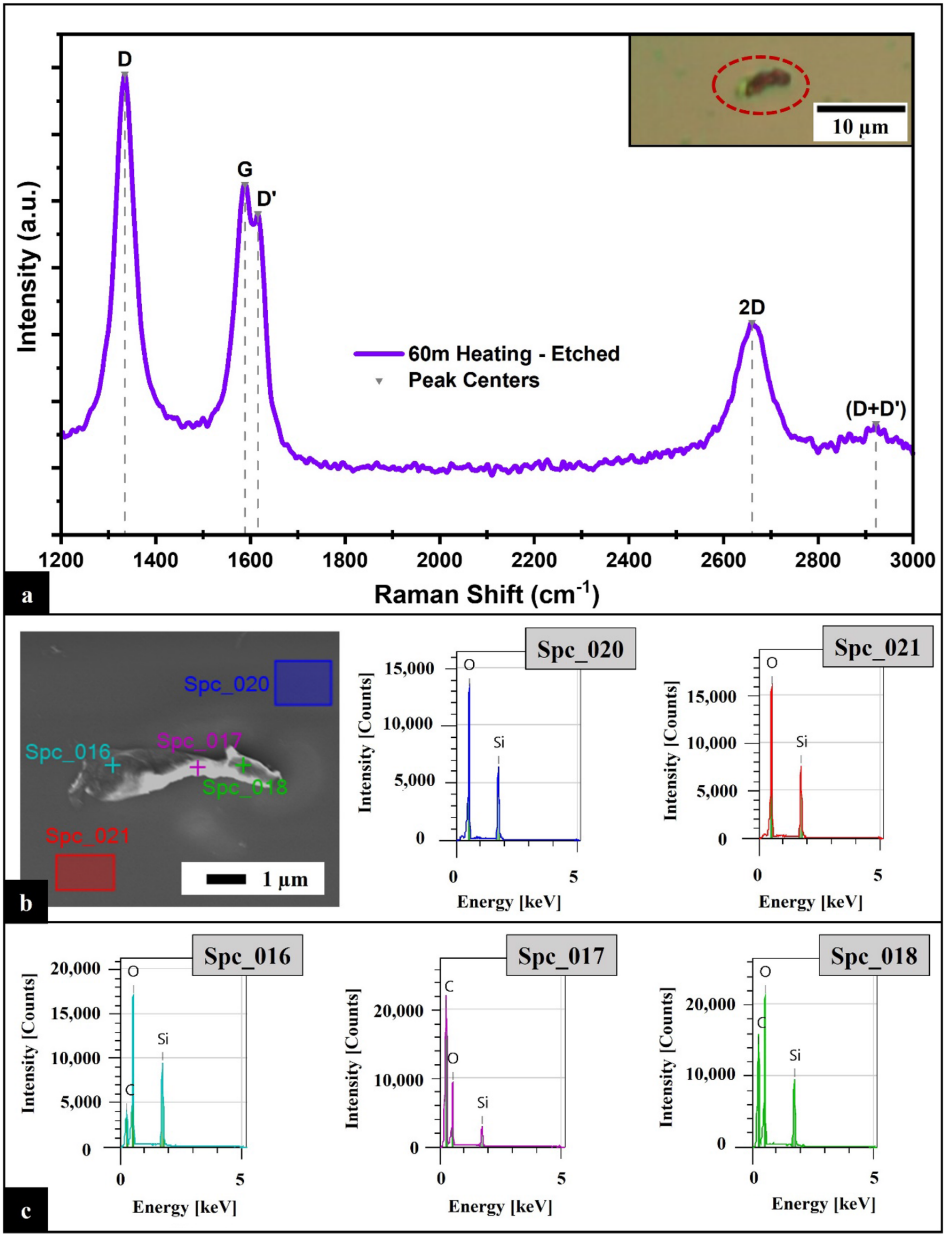
(**a**) Raman spectrum of the sample directly on the insulating substrate after 60 min of heating and etching, inset of the optical image. (**b**) SEM indicating the points and surfaces studied under EDS with the compositional analysis results of surface areas Spc_020 and Spc_021. (**c**) Compositional analysis results of points Spc_016, point Spc_017, and point Spc_018, respectively.

To produce MLG on the insulating substrate, the sample is heated for 60 min. The sample is then etched for approximately 24 h in 10% Nitric Acid (HNO_3_) to remove the Sn, any a-C, and the synthesized structure on the metal (Fig. 3, Step 6). The Raman spectra obtained from this region (Fig. 5a) are composed of well-defined peaks, indicating the formation of MLG on the SiO_2_ substrate. The 2D peak at 2660 cm^-1^ with an estimated FWHM of 86 cm^-1^ and the I_G_/I_2D_ ratio of 1.65 are consistent results for MLG^29,33,36–38^. The presence of the D peak at 1335 cm^-1^ with a FWHM of 55 cm^-1^, the evident D’ peak at 1616 cm^-1^, the (D + D’) peak at 2922 cm^-1^ with a FWHM of 200 cm^-1^, and the I_D_/I_G_ ratio of 1.31 are indicative of moderate defect density, damage, and disorder^34,39–42^.

The surface area of the resultant MLG on the insulating substrate is ≈10.43 µm^2^ with ≈5 µm length (Inset Fig. 5a and b). The chemical composition, evaluated using EDS (Fig. 5b), of various points and areas (Fig. 5c) shows the presence of carbon, oxygen, and silicon (the carbon Atom% is in Supplementary Information, section “Carbon Atomic Percentage”). There is no evidence of Sn in the area, confirming the Sn was removed effectively. Furthermore, the presence of oxygen and silicon corresponds to the substrate, SiO_2_ (Fig. 5b for surface areas Spc_020 and Spc_021).

The irregular shape of the MLG areas (Inset of Figs. 4a and 5b) can be attributed to several factors. The melting of the metal and the consequent cooldown during this time period change the morphology of the surface. It is also possible that the metal’s thickness is not uniform throughout the entirety of the surface. Additionally, heat dissipates vertically and laterally, which increases the area of interaction and therefore increases the surface of the MLG. Furthermore, utilizing a mechanical pump and lack of active vibration isolation could introduce undesired vibration into the system, causing unstable and inconsistent contact between the tip and the surface of the sample, therefore increasing the area of heating.

## Conclusions

In this study, a new method is presented to synthesize MLG directly and locally at low temperatures. Utilizing a resistive nanoheater probe, amorphous carbon is heated to 250 °C to form MLG at the surface of the Sn film and directly on the insulating substrate. The Raman spectra show the formation of MLG after heating for 5 min. Furthermore, Raman results indicate that after locally heating for 60 min and subsequent etching, MLG is synthesized directly on the insulating substrate.

The MLG achieved here is from 3 to 5 layers^3,26,29,33^. The quality, size, and even the number of graphene layers of the MLG film can be further optimized by adjusting synthesis parameters, such as layer thicknesses, pressure, flow, heating time, and temperature. We believe it is possible to generate graphene monolayers with further tuning of different synthesis parameters such as the metal thickness, metal deposition, a-C thickness and type of deposition, heater contact area, and vibration isolation.

The ability to directly synthesize MLG at locations of interest on a substrate at lower temperatures makes in-situ MIC a convenient method for synthesis onto metals and substrates. Directly growing graphene onto the surface of electrodes provides protection from oxidation and corrosion, allowing the inclusion of out-of-vacuum micro/nanofabrication steps into three-dimensional architectures. Additionally, this method could be applicable to other group IV semiconductor materials and metal catalyst combinations such as Si-Al, Si-Ag, Ge-Al, Ge-Ag, and C-Ni^43–48^. Moreover, throughput can be significantly improved using thermal probe tip arrays^49^, and for uniform heating or for different applications that require larger areas, other probe designs can be implemented, such as flat-shaped tips^50^. Overall, we believe the presented method permits inexpensive, fast, and controlled fabrication of graphene-based devices, interconnects, and electrodes for widespread micro/nanoelectronics applications.

## Supplementary Information

Supplementary Information.

## Data Availability

All data related to this paper can be requested from the corresponding authors upon reasonable request.
