# Supplementary material for: Selective area multilayer graphene synthesis using resistive nanoheater probe
Source: Sci Rep. 2023 May 17;13:7976. doi: 10.1038/s41598-023-34202-y (PMC10192444; doi:10.1038/s41598-023-34202-y)
Supplement: Supplementary file 1 — Supplementary Information. [file 41598_2023_34202_MOESM1_ESM.pdf]

Supplementary Information:
Selective Area Multilayer Graphene Synthesis Using Resistive Nanoheater Probe

Ingrid Torres[1*], Sadegh Mehdi Aghaei[2], Nezih Pala[1], and Angelo Gaitas[3*]

[1]Department of Electrical and Computer Engineering, Florida International University, Miami, FL 33172.
[2]Department of Mechanical Engineering, Worcester Polytechnic Institute, Worcester, MA 01609.
[3]Icahn School of Medicine at Mount Sinai, New York, NY 10029.
[*]I. Torres; itorr001@fiu.edu
[*]A. Gaitas; angelo.gaitas@mssm.edu

Resistive Nanoheater Probe Top View

The figure below outlines the top views/mask layouts of the most relevant steps and is complementary to Fig. 2. Figure S.1a highlights the top view of the tip formation by performing photolithographic patterning and potassium hydroxide (KOH) etching. Figure S.1b displays the layout used to pattern the cantilever on the front through Deep Reactive Ion Etch (DRIE), followed by depositing chrome and gold to make the heaters and contacts (Fig. S.1c). Figure S.1d shows the layout used to pattern the back of the substrate executing DRIE to shape the cantilever.

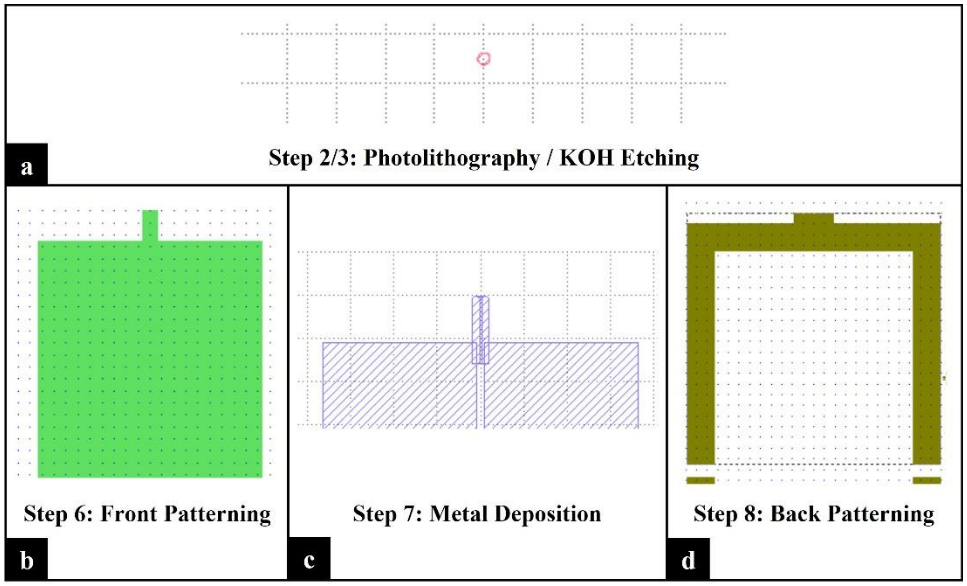

Supplementary Figure S.1. Mask layouts of the resistive nanoheater probe showcasing the top views for the process steps: (**a**) Photolithography followed by KOH etching, (**b**) front pattering through DRIE, (**c**) metal deposition for pads and heater, (**d**) back pattering through DRIE.

Resistive Nanoheater Probe Calibration

The figure below (Fig. S.2) shows the arrangement used to calibrate the resistive nanoheater probe. The 12 µm thermocouple (CHAL0005, Omega Engineering, Norwalk, CT, USA) is mounted in a fixed position on top of a metallic hand with a magnetic base. The resistive nanoheater probe is mounted on a soft sponge placed on top of a 3-axis manual micromanipulator with a magnetic base. The micromanipulator allows movement of the probe in the x, y, and z directions. First, the probe and the thermocouple are placed close enough until they are visible through the camera. Then, the probe is connected with alligator clips to a source meter (Keithley 2400, Tektronix, Inc., Beaverton, OR, USA) to feed voltage to the resistive nanoheater probe. Once everything is ready, they are zoomed in until the thermocouple is clear on the screen. The probe is then moved through the micromanipulator in all the necessary directions to make contact with the thermocouple. With the source meter, the voltage is increased to raise the temperature of the tip, and its value is recorded, as shown in Fig. 1b.

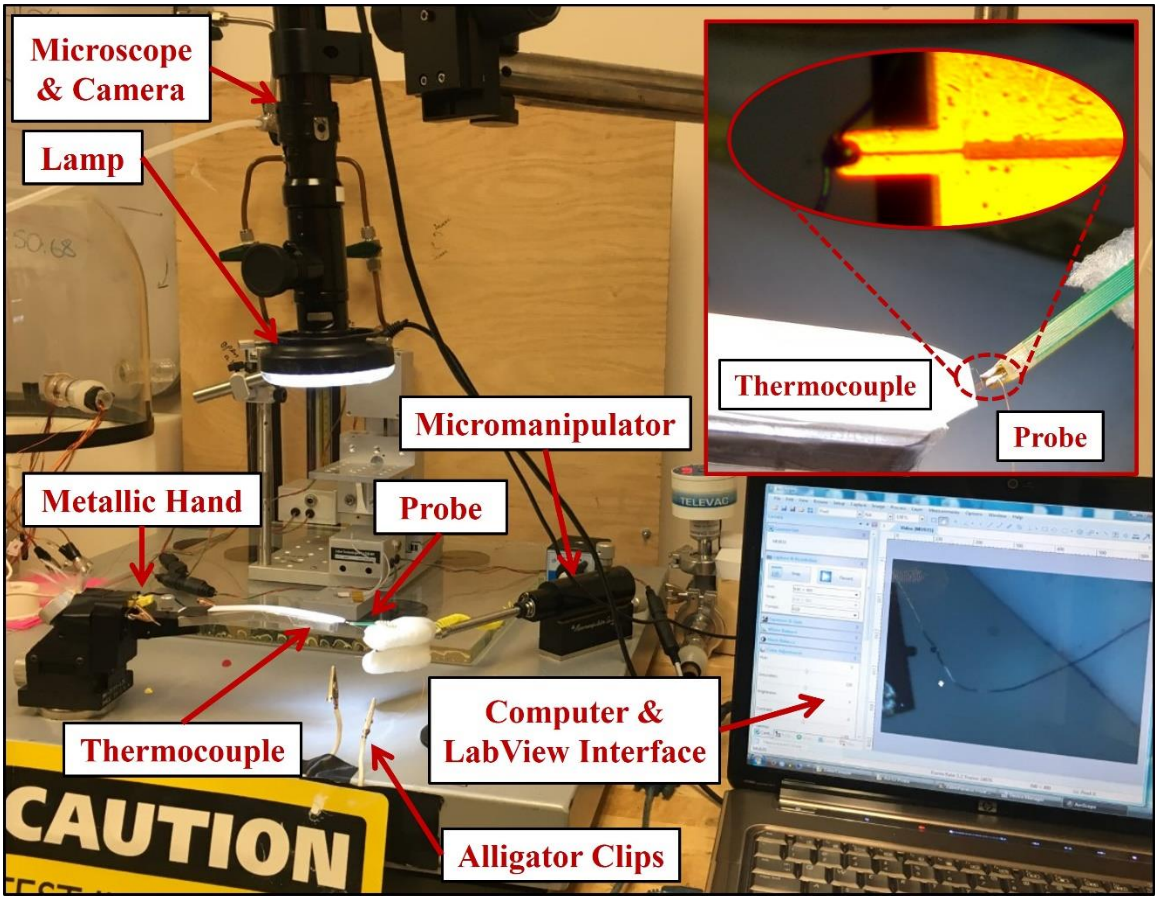

Supplementary Figure S.2. Resistive nanoheater probe temperature calibration setup with the thermocouple on metallic hand. Inset of an enhanced view of the probe in contact with the thermocouple for temperature measurement.

Experimental Setup

The figure below shows the schematics of the homemade system for multilayer graphene in-situ synthesis, detailed in the methodology section of the paper.

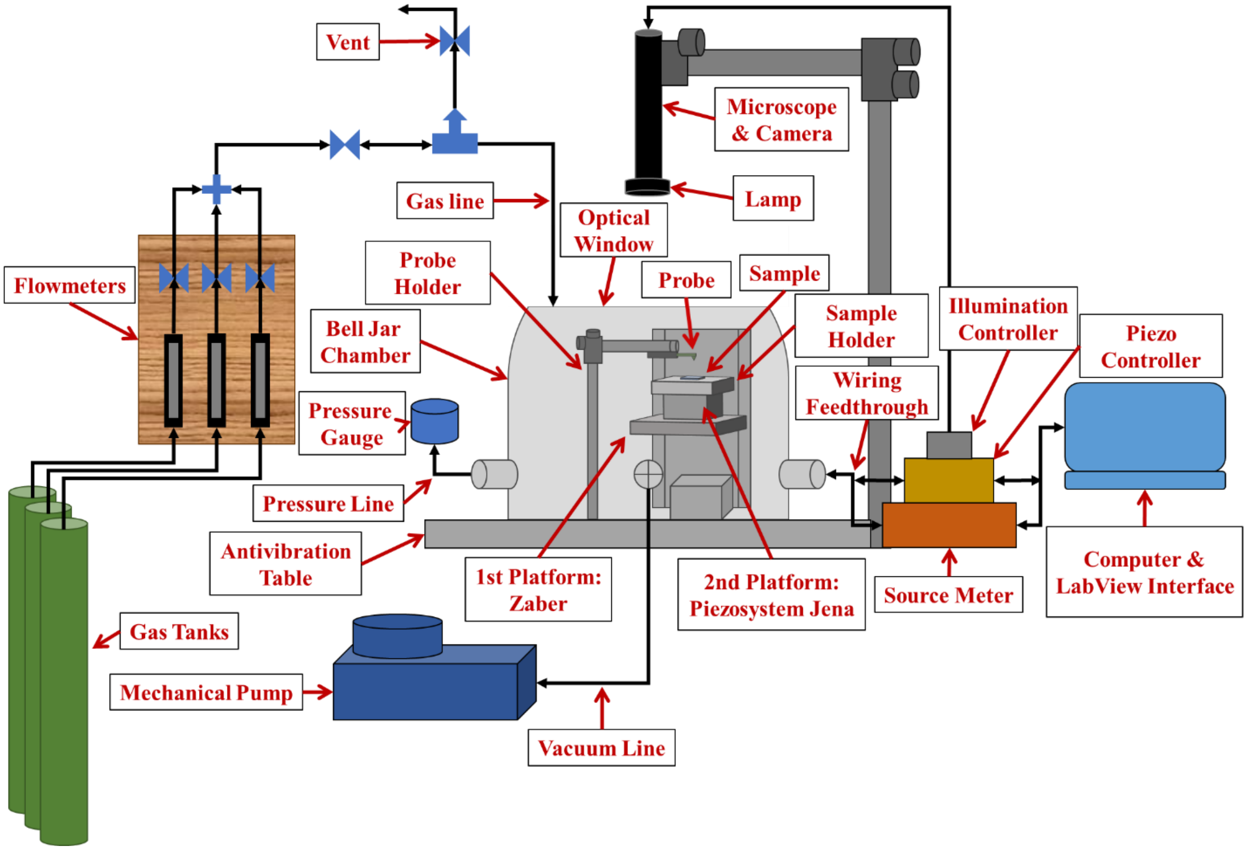

Supplementary Figure S.3. Homemade system schematic for in-situ synthesis detailing each component of the system. The system consists of a tailored glass chamber with a flat top to be used as an optical window. Each fitted connector of the chamber serves a purpose, such as pressure monitoring, bidirectional gas flow, vacuum, and electrical feed. A fixture and a movable stage comprise the interior of the chamber in order to hold the resistive nanoheater probe and to maneuver a sample in the XYZ direction, respectively. The LabView program in the computer allows the user to control the platforms for precise movement of the sample to touch the probe for synthesis.

Raman Spectra Evolution

The Raman spectra of the samples heated at different times are shown in Figure S.4. Peak fitting with Lorentzian function modeling is used to estimate the Full Width at Half Maximum (FWHM). Stage 1 and stage 2 of the Ferrari-Robertson (FR) Model are used to analyze disorder on graphene[1], known as low-disorder and high-disorder regimes, respectively[2].

Once the area of interest is locally heated for one minute, the separation between the D and G peaks becomes noticeable in the Raman spectra, as seen in Fig. S.4b. The G peak shifts towards a higher value, 1578 cm^{-1}, and the intensity of the D peak shifts to 1352.12 cm^{-1}. When a-C is heated after deposition, it follows an ordering trajectory from a-C to graphite following stage 2 in the FR model[3]. In the presence of thermal energy, the clustering of sp2 sites increases into ordered aromatic rings, and the average distance C=C shortens[3,4]. The intensity D-to-G ratio (I_D/I_G) obtained in this phase is 0.80. The appearance of the 2D peak indicates graphitic development for which the intensity G-to-2D ratio (I_G/I_{2D}) is 1.74. At this stage, the D and G peaks show to have FWHM of ≈335 cm^{-1} and ≈105 cm^{-1}, respectively. The 2D peak band measures an FWHM of ≈85 cm^{-1}.

After continuous heating of 15 minutes, the intensity of the D peak in the Raman spectrum rises at almost the same level as the intensity of the G peak, as seen in Fig. S.4d, consequently increasing the I_D/I_G ratio to 0.97. On the other hand, the I_G/I_{2D} ratio is calculated to be 1.50. However, the D peak does not shift much, remaining at 1333 cm^{-1}, whereas the G peak shifts upward to 1584 cm^{-1}, and the 2D peak shifts downward to 2662 cm^{-1}. Furthermore, the D' peak subtly appears ≈1620 cm^{-1} as a shoulder with the G peak that is hardly detected. The I_D/I_G ratio increases and the strengthening of the D peak indicates an increase in the number of defects[5,6]. The possible activation of the D' peak suggests an amorphization trajectory, namely disordering, in the Raman spectrum of the FR Stage Model[1]. Additionally, the G and 2D peaks slightly increase their width while the D peak is decreased, showing FWHM of 83 cm^{-1} for the D band, 85 cm^{-1} for the G band, and 87 cm^{-1} for the 2D band. Between the D and G peaks, there is an unidentified small peak that is irrelevant to the MLG structure characterization, and therefore its origin is not examined. The Raman spectra of virgin Sn show characteristic peaks below 700 cm^{-1} and no presence of peaks above this value[7]. However, it has been revealed that the Raman spectrum for SnO develops other peaks when annealed, but no study has been found showing peaks beyond 700 cm^{-1} [8]. Therefore, the peak in between the D and G may come from the metal when annealed at 250 °C or a yet to be identified source.

When the sample is heated for 60 min, not only the D, G, and 2D peaks intensify, but they also widen, as shown in Fig. S.4e. First, the D peak maximum intensity surpasses the intensity of the G peak, consequently increasing the I_D/I_G ratio to 1.10. Additionally, I_G/I_{2D} ratio is found to be equal to 1.94. The D and the 2D peaks shift downward to 1327 cm^{-1} and 2644 cm^{-1}, respectively. Meanwhile, the G peak remains at 1581 cm^{-1}. The FWHM of the peaks are ≈95 cm^{-1} for the D band, ≈105 cm^{-1} for the G band, ≈118 cm^{-1} for the 2D band, confirming the increase of their width. The broadening of the G peak is attributed to the intensification of the D' peak, which merges with the G peak. The activation of the D' peak is due to defects causing intravalley double-resonance process[9]. For the sake of simplicity, usually, these two peaks are conveniently considered as one broad single G peak when the separation between them is not apparent[10]. Moreover, an additional visible peak appears ~ 2900 cm^{-1} with an FWHM of ≈200 cm^{-1}, which

corresponds to the (D + D’) peak[9,10]. The resultant Raman peaks at 60 minutes confirm that the spectra evolution follows the FR Model stage 1 disorder[1,3,10]. Therefore, the continuous heating of the region breaks the crystal symmetry, increasing the defect density and introducing disorder into the MLG. It is worth noting that disorder can also be induced by sp3-defects which usually originate from chemisorptions, also referred to as chemical defects, where there is a change of hybridization (from sp2 to sp3) such as oxidation[11–13].

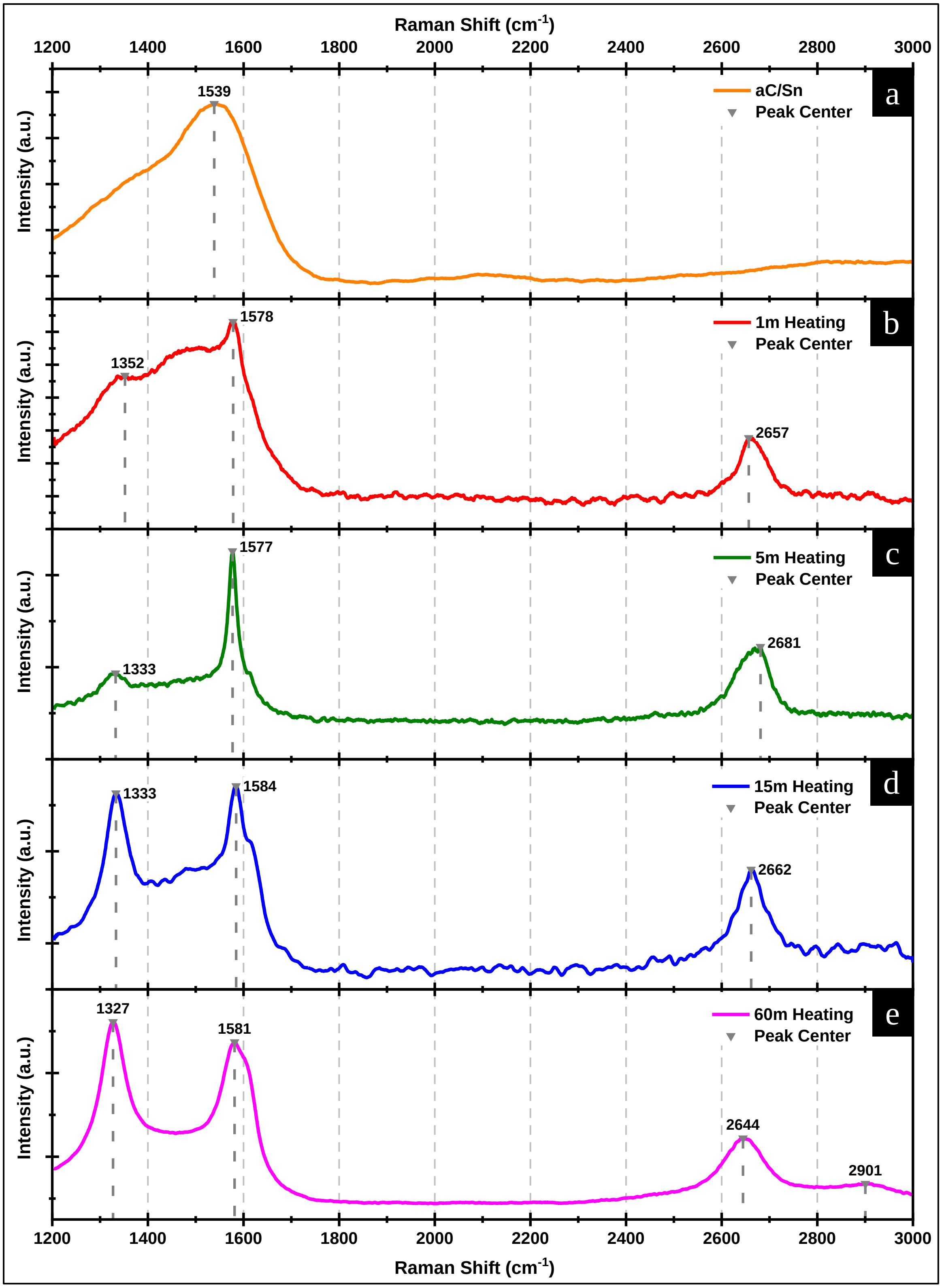

Supplementary Figure S.4. Raman spectra time evolution of the sample for different heating times. (**a**) The Raman spectrum of the sample after a-C deposition. The Raman spectra of sample after being heated for: (**b**) 1 minute, (**c**) 5 minutes, (**d**) 15 minutes, and (**e**) 60 minutes.

SEM-EDS of Amorphous Carbon

The following figure (Fig. S.5a) shows the surface area after amorphous carbon is sputtered on top of tin (Sn). Energy Dispersive X-ray Spectroscopy (EDS) is used to evaluate its composition and the results are shown in Fig. S.5b. The presence of carbon, tin, and oxygen are detected through the EDS. The carbon and tin correspond to the deposited amorphous carbon (a-C) and Sn layer. Moreover, the detection of oxygen may come from the surface of the metal or from the SiO_2 substrate. The silicon is not detected here due to the penetration of the electron beam not reaching the necessary depth. The resultant carbon atomic percentage (Atom%) is 82.63.

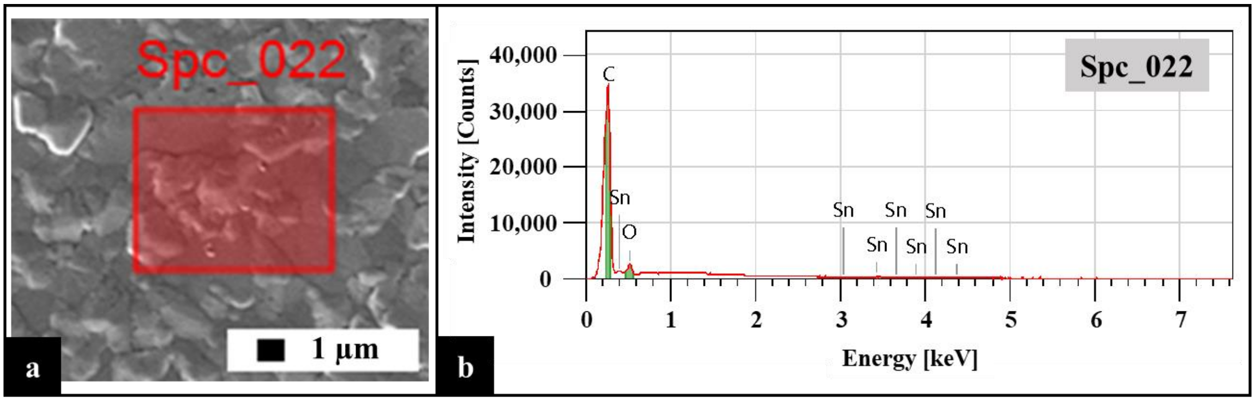

Supplementary Figure S.5. (**a**) SEM image with red square (Spc_022) indicating the surface studied under EDS after a-C deposition. (**b**) EDS spectrum result of surface area Spc_022.

Carbon Atomic Percentage – Figure 5.c

The carbon atomic percentages (Atom%) from the analysis are 16.97 for Spc_016 point, 61.41 for Spc_017 point, and 37.34 for Spc_018 point.

REFERENCES:

1. Ferrari, A. C. Raman spectroscopy of graphene and graphite: Disorder, electron–phonon coupling, doping and nonadiabatic effects. *Solid State Communications* **143**, 47–57 (2007).
2. Beams, R., Gustavo Cançado, L. & Novotny, L. Raman characterization of defects and dopants in graphene. *J. Phys.: Condens. Matter* **27**, 083002 (2015).
3. Ferrari, A. C. & Robertson, J. Interpretation of Raman spectra of disordered and amorphous carbon. *Phys. Rev. B* **61**, 14095–14107 (2000).
4. Adelhelm, C., Balden, M., Rinke, M. & Stueber, M. Influence of doping (Ti, V, Zr, W) and annealing on the sp2 carbon structure of amorphous carbon films. *Journal of Applied Physics* **105**, 033522 (2009).
5. Li, X. *et al.* Large-Area Synthesis of High-Quality and Uniform Graphene Films on Copper Foils. *Science* **324**, 1312–1314 (2009).

6. Merlen, A., Buijnsters, J. & Pardanaud, C. A Guide to and Review of the Use of Multiwavelength Raman Spectroscopy for Characterizing Defective Aromatic Carbon Solids: from Graphene to Amorphous Carbons. *Coatings* **7**, 153 (2017).
7. Lafuente, B., Downs, R. T., Yang, H. & Stone, N. The power of databases: the RRUFF project. In: Highlights in Mineralogical Crystallography, T Armbruster and R M Danisi. https://rruff.info/Tin.
8. Nikiforov, A. *et al.* Formation of SnO and SnO2 phases during the annealing of SnO(x) films obtained by molecular beam epitaxy. *Applied Surface Science* **512**, 145735 (2020).
9. Elias, D. C. *et al.* Control of Graphene's Properties by Reversible Hydrogenation: Evidence for Graphane. *Science* **323**, 610–613 (2009).
10. Ferrari, A. C. & Basko, D. M. Raman spectroscopy as a versatile tool for studying the properties of graphene. *Nature Nanotech* **8**, 235–246 (2013).
11. Eckmann, A. *et al.* Probing the Nature of Defects in Graphene by Raman Spectroscopy. *Nano Lett.* **12**, 3925–3930 (2012).
12. Felten, A., Eckmann, A., Pireaux, J.-J., Krupke, R. & Casiraghi, C. Controlled modification of mono- and bilayer graphene in O_2 , H_2 and CF_4 plasmas. *Nanotechnology* **24**, 355705 (2013).
13. Wu, J.-B., Lin, M.-L., Cong, X., Liu, H.-N. & Tan, P.-H. Raman spectroscopy of graphene-based materials and its applications in related devices. *Chem. Soc. Rev.* **47**, 1822–1873 (2018).
